# The Importance of Patient-Centered Research in the Promotion of Postpartum Mental Health

**DOI:** 10.3389/fpsyt.2021.720106

**Published:** 2021-09-16

**Authors:** Ariana M. Albanese, Pamela A. Geller, Christina A. Sikes, Jennifer L. Barkin

**Affiliations:** ^1^Women's Health Psychology Laboratory, Department of Psychology, Drexel University, Philadelphia, PA, United States; ^2^Houston Country Health Department, Georgia Department of Public Health, Warner Robbins, GA, United States; ^3^Department of Community Medicine, Mercer University School of Medicine, Macon, GA, United States

**Keywords:** patient-centered, postpartum, mental health, behavioral health, women's health

## Introduction

Though individuals, foundations, and governments have economically invested in improving maternal-child health for decades, the advancement of newborn and family health outcomes has proved elusive in multiple regions of the globe (1).^1^ Additionally, though researchers often seek the perspective of pregnant and postpartum patients concerning their experience of healthcare or specific models of care (2), the maternity care research issues which mothers believe ought to be prioritized are understudied (3). There is also substantial evidence of a mismatch between postpartum healthcare offerings and the support desired by mothers, particularly with regard to a relative neglect of the maternal psychosocial experience (4–7). It thus is becoming increasingly clear that mothers' experiences must be central to research aimed at improving maternal postpartum mental health. This opinion article therefore echoes calls for increased patient-centered research efforts in postpartum care (8), while emphasizing the value of this mode of inquiry for the promotion of maternal *mental* health specifically.

## Gap Between What Mothers Want Out of Postpartum Care and What Is Offered

There is a documented gap between what mothers desire in the postpartum period, and what is offered, with content tending to favor topics like infant care while mother's psychosocial adjustment is relatively neglected. For example, Guerra-Reyes and colleagues found that postpartum health information gaps (i.e., instances in which mothers felt they were not equipped with the health information they desired) were common, particularly in the topic areas of sexual and mental health (5). Henshaw et al. found similar results, reporting that postpartum mothers desired reliable healthcare information on parental adjustment and maternal health, as most participants reported that their providers focused on infant needs rather than maternal mental and physical health needs, leaving mothers feeling unprepared to care for themselves (9). Barkin et al. similarly found that postpartum mothers struggle to perform self-care (10). A recent qualitative study conducted with both postpartum mothers and nurses suggested that there is a disconnect between the expectations and priorities of the two parties with regard to postpartum care (4). While nurses emphasized completing standardized pre-discharge education on topics such as newborn care and safety in the immediate postpartum period, mothers found that it was difficult to effectively engage with this intervention given the many stressors and distractions commonly encountered during the immediate postpartum. The authors concluded that this standardized education may detract from mothers' priorities such as connection to family and care team as well as rest (4). Another mixed methods study seeking to elucidate the healthcare needs that mothers identified in the postpartum period, found that the participants had a wider conception of healthcare needs than that which is typically addressed, including the area of social support (7). Martin and colleagues conducted focus groups with both clinical providers (i.e., obstetricians and midwives) as well as mothers, and similarly found that the major postpartum-related concerns held by clinicians differed from those held by mothers, and that many mothers did not feel prepared for the postpartum transition (11). Specifically, Martin et al. found that mothers were concerned about how postpartum symptoms would affect daily functioning while providers focused on potentially dangerous physical health conditions like infection. Additionally, while mothers looked to their providers for support on psychosocial concerns (e.g., linking them to additional resources like lactation specialists and social workers) clinicians felt that they lacked the training and time to address psychosocial concerns (11). While the American College of Obstetricians and Gynecologists (ACOG) recommends screening for postpartum depression as part of comprehensive postpartum follow-up (12), mood and emotional well-being comprise but one of nine domains recommended to be covered during postpartum visits. The other recommended domains focused on infant care and maternal physical health and recovery, suggesting that mental health concerns may not receive the time and attention required to fully address them. Additionally, mental health screening is not implemented universally and is sometimes implemented in a way that is inconsistent or not based on recommended guidelines (13). It has been estimated that less than half of the mothers with postpartum depression are identified by providers and that most individuals do not seek help (14–17).

## Importance of Patient-Centered Care to Postpartum Mental Health

It is perhaps not surprising that there are maternal postpartum mental health ramifications resulting from this gap in care. First, as described above, it can leave mothers feeling unprepared for the challenges of the postpartum transition. This feeling of unpreparedness can lead to feelings of overwhelm and other negative mental health consequences (18). Additionally, when mothers are not connected with sufficient and appropriate support in the postpartum period, this can confer multiple negative side effects on mother and baby, including negative maternal emotional responses such as anxiety, stress, and loneliness (19). Maternal distress in turn is associated with impairments in maternal-infant interactions and caregiving practices (20), as well as impaired outcomes for the infant such as psychopathology and developmental issues (21, 22). A recent article presenting a framework to guide clinical care and research for perinatal mood and anxiety disorders (PMADs) identified the psychosocial process of achieving “matriescence,” or “becoming a mother” as one of the key focus areas (23). As the authors argue, in order to help mothers address this transition successfully, researchers and clinicians must understand the lived experience of the mothers they are seeking to support.

Thus, given the mental health risks of ineffective postpartum care as well as the importance of understanding mothers' lived experiences to define effective care, centering the patient voice in research efforts to improve maternal postpartum mental health appears critical. Not to mention, as Daly et al. argued, it is likely that conducting research on the issues that are most important to postpartum mothers will confer the greatest benefit to them (3). Relatedly, it is important to include representatives from diverse backgrounds in patient-centered research to ensure both that the patient voices accurately reflect the population for whom this research will serve and to confer maximum generalizability. Further, given the racial disparities in perinatal mental health (24), the increased incidence of mistreatment during pregnancy and childbirth for women of color (25), and the startling racial disparity in severe maternal morbidity (26) it is especially important to include Black, Indigenous, and people of color (BIPOC) in this work.

Patient-centered care focuses efforts on patient needs, beliefs, and preferences, as opposed to provider-centered care which preferences the needs of clinical providers. As such, patient-centered research requires active participation of patients as key stakeholders (27, 28). Some perinatal researchers have readily adopted a patient-centered approach. For example, Verbiest et al. brought together mothers, healthcare providers, and advocates as part of the *4th Trimester Project*, which sought to better understand maternal postpartum needs (29). This study was designed with careful attention to the needs of mothers and infants, patient stakeholders were present throughout project meetings, and patient feedback and criticism was solicited throughout the study. Thus, Verbiest et al. provide a great example of truly centering the patient voice in the research process. Additionally, Barkin and colleagues utilized qualitative work with mothers in the development of the Barkin Index of Maternal Functioning, producing a patient-centered measurement metric of maternal well-being (30). Also, Albanese and colleagues built off of the work of Barkin et al. by utilizing qualitative inquiry to identify the factors which are most influential to maternal postpartum functioning (31). There also has been important patient centered work done that specifically centers on racial disparities. The work of Altman and colleagues provides suggestions that mothers indicated would help to build trust between patient and provider and thereby improve their experience of healthcare (32). McLemore and colleagues' work focus group work also added to the growing literature base demonstrating the racism, discrimination, and disrespect women of color experience in their perinatal healthcare encounters (33).

Previous clinical research has demonstrated that patient-centeredness is important to effective mental healthcare. For example, regarding adherence to antidepressant medication, it is now understood that each patient's unique preferences, beliefs, and experiences are key to understanding how (and if) medication is taken (34). A study conducted in the perinatal population and examining concerns around antidepressant use indicated a need for increased involvement of patients in treatment decision making (35). When patients are actively involved in medical decision-making, this has been shown to improve outcomes for patients with depression (36) whereas the assignment of an unacceptable depression treatment has been shown to lead to negative outcomes like poor treatment alliance and high rates of attrition (37). Several other non-pharmacological interventions for peripartum distress that centered the patient by taking an individualized approach and making efforts to lower barriers to support have also demonstrated a positive impact on mental health outcomes (38, 39).

Importantly, professional organizations have also recently put forth recommendations that move toward more patient-centered protocols, such as the previously mentioned guidelines from ACOG for comprehensive postpartum care as well as the Association of Women's Obstetric Health and Neonatal Nursing's Postpartum Discharge Education Program which seeks to better educate all women, regardless of risk, on potential life-threatening postpartum complications thereby encouraging them to seek care in a timely fashion and helping them to advocate for their care more effectively (12, 40). Also of note, the ACOG recommendations call for postpartum care to be a continuous process that begins with contact with a patient's OB-GYN at 3 weeks postpartum, is tailored to individual needs, is continued with ongoing care as needed, and is bookended with a comprehensive exam at 12 weeks postpartum (12). These recommendations also push for reimbursement policies that view postpartum care as an ongoing process rather than an isolated visit. This is an important first step as it demonstrates acknowledgment from the healthcare sector of the complexity of the postpartum transition, rather than signaling that becoming a mother is “natural” or “easy” and thus only requires one 6-week postpartum medical visit. However, despite such efforts, a recent rapid review on patient-centered care for women found that there is room for growth, particularly in the domain of responding to patient emotions, as much extant work has focused on the exchange of information from provider to patient during healthcare encounters rather than attending to the emotional support needs of patients during such visits (41). This is significant, as previous work has found that women have an expectation for psychosocial support from healthcare providers in the postpartum and desire reassurance around postpartum symptoms (11).

## Conclusion and Future Directions

The postpartum transition is a major life adjustment that can challenge mothers' mental health. As the experts on their experience, mothers ought to be actively involved in the direction of research and clinical care in the postpartum period. Further, previous work has demonstrated the positive impact of patient-centered care on mental health outcomes. However, a gap remains between what providers recommend in the postpartum period and what mothers desire. Thus, in order to ensure the psychological health of mothers and infants, it is critical that maternal voices of diverse backgrounds be centered in clinical research efforts targeting the postpartum period. It is our hope that this article will inspire more clinical researchers, particularly those targeting maternal postpartum mental health, to center the patient voice in research efforts in order to better address mothers' psychosocial needs throughout the postpartum transition.

## Author Contributions

AA and PG: conceptualization. AA: writing—original draft preparation. AA, PG, JB, and CS: writing—review and editing. All authors have reviewed the manuscript and agree to this version.

## Conflict of Interest

The authors declare that the research was conducted in the absence of any commercial or financial relationships that could be construed as a potential conflict of interest.

## Publisher's Note

All claims expressed in this article are solely those of the authors and do not necessarily represent those of their affiliated organizations, or those of the publisher, the editors and the reviewers. Any product that may be evaluated in this article, or claim that may be made by its manufacturer, is not guaranteed or endorsed by the publisher.

## Footnotes

^1^Although we will use gendered language in this manuscript, this topic applies to anyone who can become pregnant including cisgender women as well as trans men and non-gender binary individuals.
